# Case Report and Literature Review Illustrating the Clinical, Endoscopic, Radiologic, and Histopathologic Findings with Prepouch Ileitis after IPAA and Restorative Proctocolectomy for Refractory Ulcerative Colitis

**DOI:** 10.1155/2018/7506069

**Published:** 2018-07-30

**Authors:** Christienne Shams, Seifeldin Hakim, Mitual Amin, Mitchell S. Cappell

**Affiliations:** ^1^Division of Gastroenterology & Hepatology, Department of Medicine, William Beaumont Hospital, 3535 W. Thirteen Mile Rd, Royal Oak, MI 48073, USA; ^2^Department of Pathology, William Beaumont Hospital and Oakland University William Beaumont School of Medicine, 3601 W Thirteen Mile Rd, Royal Oak, MI 48073, USA; ^3^Division of Gastroenterology & Hepatology, Department of Medicine, William Beaumont Hospital and Oakland University William Beaumont School of Medicine, 3535 W. Thirteen Mile Rd, Royal Oak, MI 48073, USA

## Abstract

Prepouch ileitis (PI) is an uncommon complication of ileal pouch anal anastomosis (IPAA) and restorative proctocolectomy (RPC) for treatment of refractory ulcerative colitis (UC). A case is reported of PI in a 16-year-old girl who presented with severe UC that was initially stabilized with infliximab therapy but re-presented 1 year later with severe UC, refractory to infliximab and corticosteroid therapy, which required IPAA and RPC. Her symptoms resolved postoperatively, but she re-presented 1 year later with 10 loose, bloody, bowel movements/day and involuntary 6-Kg weight-loss. Computerized tomographic enterography showed focal narrowing and mucosal enhancement of the pouch and focal narrowing, abnormal mucosal enhancement, and mural thickening of the prepouch ileum. Pouchoscopy revealed exudates and ulcerations in both the pouch and prepouch ileum up to 50 cm proximal to pouch, as confirmed by histopathology of pouch and ileal biopsies. Capsule endoscopy revealed no small intestinal lesions beyond 50 cm from the pouch. She required antibiotics, hydrocortisone enemas, and eventually azathioprine to control her symptoms. She remains asymptomatic 4 years later while chronically administered azathioprine therapy. Comprehensive literature review demonstrates that this case illustrates the classical clinical, radiologic, endoscopic, and histopathologic findings in PI, a relatively rare syndrome.

## 1. Introduction

Restorative proctocolectomy (RPC) and ileal pouch anal anastomosis (IPAA) are the treatment of choice for ulcerative colitis (UC) refractory to medical therapy, for UC with severe dysplasia or colon cancer, and sometimes for familial adenomatous polyposis [1–4]. This surgery becomes necessary in 10-30% of patients within one decade after diagnosis of UC [1]. Outcomes are generally favorable, lasting up to 20 years [4]. Prepouch ileitis (PI) affects about 4% of patients undergoing RPC and IPAA [2, 5]. A case is reported of PI after RPC and IPAA, illustrating the classical clinical, radiologic, endoscopic, and histopathologic findings of this rare syndrome.

## 2. Case

**Figure 1:**
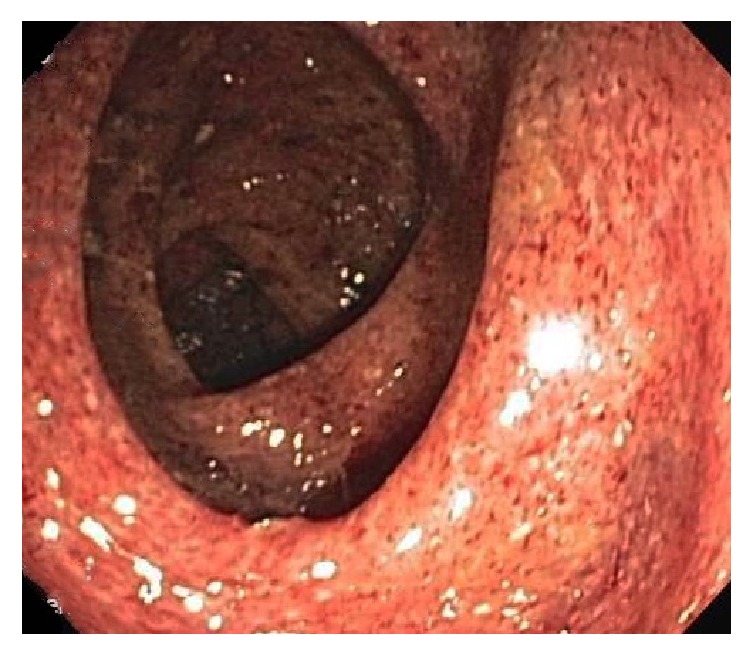
Colonoscopy with intubation of terminal ileum shows diffusely erythematous and granular mucosa with focal exudation affecting the rectum through ascending colon, with sparing of the cecum and terminal ileum. The endoscopic findings are consistent with UC.

**Figure 2:**
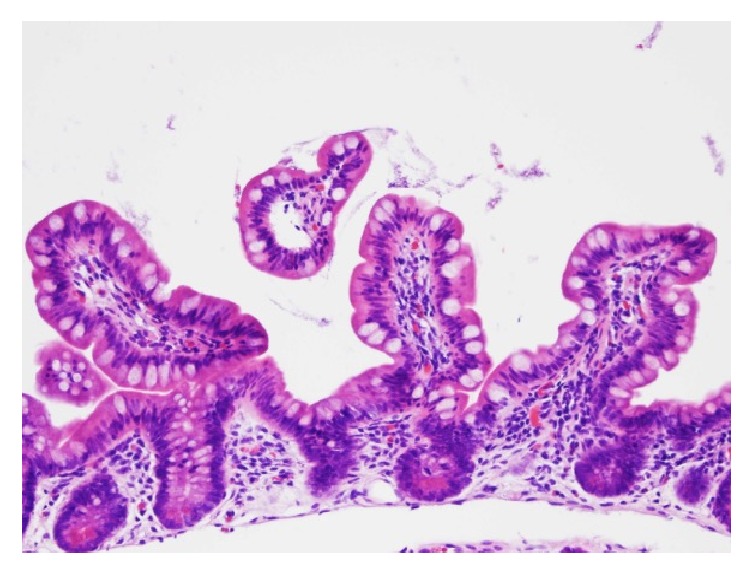
Photomicrograph of histopathology with H&E stain of biopsy specimen shows normal ileal mucosa prior to postoperative occurrence of prepouch ileitis, with relatively normal villous height and preserved villous-to-crypt ratio.

**Figure 3:**
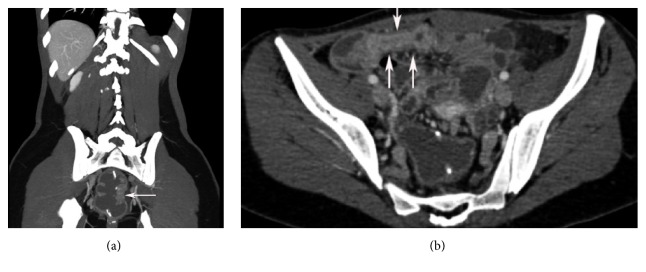
(a) Coronal section of computerized tomographic enterography shows focal mural thickening and enhancement of afferent limb of the J-pouch 1 year after undergoing IPAA and RPC for ulcerative colitis refractory to medical therapy. (b) Transverse section of computerized tomographic enterography shows mucosal hyperenhancement, mural thickening, and luminal narrowing of a 9.4 cm long segment of terminal ileum 1 year after undergoing IPAA and RPC for ulcerative colitis refractory to medical therapy.

**Figure 4:**
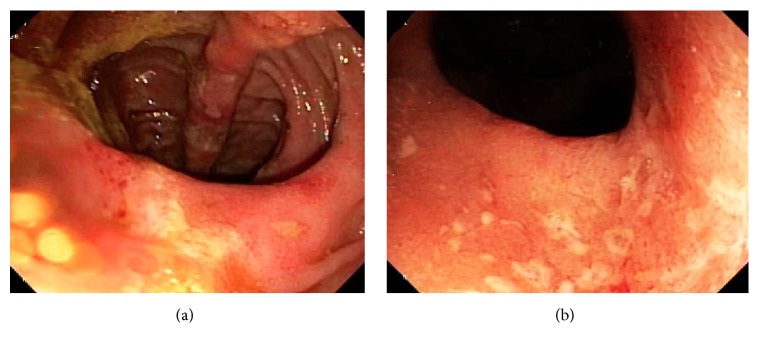
(a) Pouchoscopy shows ulceration and mild inflammation in the J-pouch. (b) Pouchoscopy shows ulceration and mild inflammation in the terminal ileum proximal to the J-pouch.

**Figure 5:**
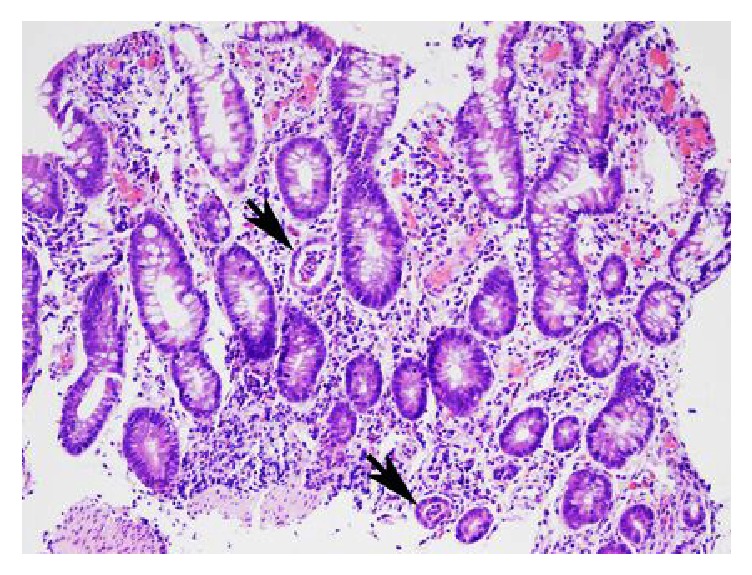
Histopathology with H&E shows near total loss of villi, crypt architectural distortion, and crypt abscesses (arrows) in endoscopic biopsies obtained 20 cm from anal verge, findings highly consistent with prepouch ileitis after creation of a J-pouch.

A 16-year-old girl with no significant past medical history presented with bloody diarrhea, abdominal cramps, tenesmus, failure to thrive, and 6-Kg weight-loss during the prior 3 months. Physical examination was unremarkable except for age-adjusted BMI at the eleventh percentile. Abdominal examination revealed a soft, nontender abdomen and normoactive bowel sounds. Laboratory analysis revealed leukocyte count=8.1 bil/L, hemoglobin=11.4 g/dL, and platelets=207 bil/L. The alkaline phosphatase is 125 U/L, with other parameters of liver function and parameters of renal function within normal limits. Colonoscopy with terminal ileal intubation revealed severely erythematous and granular mucosa with focal exudation from rectum to ascending colon, findings consistent with UC (**Figure 1**), and revealed endoscopically normal appearing cecum and terminal ileum. Histopathologic analysis of colonic biopsies revealed chronic colitis, with a moderate neutrophilic and lymphocytic mucosal infiltrate, crypt distortion, and scattered crypt abscesses. The cecum and terminal ileum appeared histologically normal (**Figure 2**). She was treated with infliximab 5 mg/Kg, with initial symptomatic relief, but re-presented 1 year later with recurrent bloody diarrhea and failure to thrive, despite compliance with infliximab therapy. She developed infliximab antibodies necessitating escalating the infliximab dose, and adding extended-release budesonide 9 mg/day and azathioprine 2 mg/kg/day (after determining that her TPMT (thiopurine methyltransferase) activity was within normal limits). Her symptoms, however, progressed despite therapeutic infliximab levels. She underwent RPC and IPAA for refractory UC, which successfully controlled her symptoms but re-presented one year postoperatively with abdominal pain, 10 loose and bloody bowel movements/day, and involuntary 5-Kg-weight-loss. Fecal lactoferrin and calprotectin levels were elevated. Stool for ova and parasites, bacterial cultures, and* Clostridium difficile* toxin A and B by polymerase chain reaction (PCR) were unremarkable. C-reactive protein (CRP) level was elevated. Computerized tomographic enterography (CTE) showed focal narrowing and enhancement of mucosa within the J-pouch and abnormal mucosal enhancement, mural thickening, and narrowing of afferent ileal limb (**Figures 3(a) and 3(b)**). Pouchoscopy showed moderate exudation and ulcerations in J-pouch (**Figure 4(a)**) and in afferent ileal limb up to 50 cm (**Figure 4(b))**. Histopathologic analysis of J-pouch and afferent ileal limb biopsies revealed chronic active inflammation, highly consistent with pouchitis and PI (**Figure 5)**. Immunohistochemistry of ileal biopsies for cytomegalovirus was negative. Capsule endoscopy revealed no small intestinal lesions more proximal than 50 cm in the afferent limb. Ciprofloxacin 500 mg twice daily and metronidazole 500 mg thrice daily were administered for the pouchitis and PI, but this treatment was subsequently escalated to include extended-release budesonide 9 mg/day and daily hydrocortisone enemas. As symptoms persisted, azathioprine 2 mg/kg/day was added, which successfully controlled her symptoms 3 months after initiating the azathioprine therapy. Six months after initiating the azathioprine therapy her fecal lactoferrin and calprotectin levels were within the normal range. At four years of follow-up, the patient has continued to be asymptomatic while chronically taking azathioprine, with normal CRP and erythrocyte sedimentation rate (ESR) levels.

## 3. Discussion

Despite lack of standard definition, PI is described as histologically evident mucosal inflammation extending beyond the reconstructed pouch up to 50 cm proximally in the afferent limb, and it is usually associated with endoscopically apparent erosions, ulcerations, erythema, and friability in the ileum that had appeared normal at endoscopy before undergoing the surgery [1, 4, 6, 7].

The incidence of PI after RPC and IPAA ranged in two large studies from 4.4% to 6% [1, 8]. PI occurs more frequently in patients who are young, who underwent early colectomy for UC, and who developed intestinal symptoms soon after undergoing RPC and IPAA, possibly because these factors are markers of biologically aggressive UC [1, 7]. Smoking cigarettes does not significantly affect the rate or severity of PI, despite smoking ameliorates UC and pouchitis [1, 2, 9]. Sex does not significantly affect the rate of PI, but males are more likely to develop pouchitis and* Clostridium difficile* infections than females [10]. Pouch anatomy may affect the risk of PI, with PI more frequently reported in W-pouches (3.4%) than in S-pouches (2.1%) or J-pouches (1.9%), even though these differences were not statistically significant [11]. PI is strongly associated with PSC in patients with UC as demonstrated by Shen et al. [1, 2, 10]. Moreover, patients with PI and PSC more likely have a concurrent autoimmune disorder, which might contribute to development of PI and pouchitis [2]. The association between PI and PSC might arise from ileal inflammation from abnormal bile acid metabolism in PSC, even though differences in ileal bile acid composition have not yet been described in PI with PSC versus without PSC [2, 9]. PI can sometimes occur in Crohn's disease (CD), but without concomitant PSC [2].

Symptoms of PI include frequent defecation, defecation difficulties, loose stools, flatus, colicky abdominal pain, GI obstruction, and involuntary weight loss [8, 11]. Pouchitis produces similar symptoms [7]. Endoscopic abnormalities with PI include erosions, ulcerations, exudates, erythema, and friability in the ileum beyond the reconstructed pouch up to 50 cm proximally in the afferent limb. Histologic analysis of endoscopic biopsies demonstrates acute or chronic inflammation. Endoscopic and histologic abnormalities in PI are similar to those in pouchitis, except that the endoscopic and histologic abnormalities extend proximally from the pouch and tend to become progressively milder proximally in PI [7, 11]. The diagnosis of PI must be initially confirmed by pouchoscopy and histology, but subsequent flares can be managed clinically, without repeating pouchoscopy [12]. PI can be diagnosed only after excluding infectious ileitis, especially from cytomegalovirus. Iwata et al. [7] reported that serum levels of interleukins (ILs), including IL-1*β*, IL-6, and IL-8, and of tumor necrosis factor-*α* are significantly elevated in both pouchitis and PI.

PI and pouchitis respond to the same therapies [1, 7]. Antibiotics and corticosteroids are the mainstays of therapy, with about 75% of patients responding to combined ciprofloxacin and metronidazole antibiotic therapy and about 25% becoming refractory to this therapy [1, 4, 7, 10]. Symptomatic response to antibiotic therapy does not necessarily guarantee mucosal healing [5]. Immunomodulators or biologic therapy is used for refractory patients [1]. PI more frequently requires escalation with immunomodulator or biologic therapy than pouchitis, possibly because PI is associated with autoimmune disorders [1, 2, 7, 10]. Infliximab has some efficacy in refractory patients, while adalimumab is used as salvage therapy in patients with adverse effects or poor response to infliximab [4, 13, 14].

This reported patient presented with many characteristics associated with PI: young age at diagnosis (18 years old), early colectomy (1 year after UC diagnosis), concurrent pouchitis, initial favorable response to RPC and IPAA surgery, and satisfactory symptomatic control of PI achieved after introducing immunomodulators. Specialized tests showed characteristic findings of PI extending to 50 cm beyond the J-pouch including exudation and ulceration on pouchoscopy; mural thickening, mucosal enhancement, and luminal narrowing of the afferent limb on CTE; and absence of lesions in the afferent limb beyond 50 cm proximally on capsule endoscopy [4]. The reported histopathology of ileal biopsies was highly consistent with PI.

PI occurs postoperatively almost exclusively in patients with UC, uncommonly in patients with CD, and extremely rarely in patients with FAP [1]. PI has been postulated to arise after RPC and IPAA for colon cancer with UC or FAP secondary to an altered and pathological ileal milieu (microflora) after surgery [4]. The pathophysiology of PI remains uncertain because of scarce data about this relatively rare condition. Moreover, PI is underreported because patients are routinely treated empirically with antibiotics for symptoms of pouch dysfunction without performing pouchoscopy, and even if pouchoscopy is performed the afferent limb is infrequently intubated [1, 7].

While some authorities believe PI represents CD misdiagnosed as UC before colonic surgery, other authorities believe PI is an extension of existing pouchitis, and still other authorities believe PI is an entirely different disease [1, 15, 16]. Given that PI can sometimes resemble CD in endoscopic appearance and clinical behavior, PI was historically misdiagnosed as CD. However, PI has recently been reported to have histologic and endoscopic characteristics distinct from those of CD [1, 13, 15]; for example, PI is limited to the terminal ileum 50 cm beyond the pouch without proximal small intestinal inflammation [4, 6, 13]. Lorenzo et al. [16] reported that 43% of cases of PI were associated with delayed diagnosis of CD in a study with prolonged postoperative follow-up averaging 20 years in patients who had been diagnosed with UC before surgery; in study patients with UC, PI was uniformly associated with pouchitis, implying a possibly shared pathophysiology of PI and pouchitis from preexistent UC, as previously reported [1, 4–6]. Bell et al. [11] reported only 50% of PI in UC patients is associated with pouchitis, and in these cases PI and pouchitis share histological and morphological similarities. These contradictory findings between Bell et al. [11] and Lorenzo et al. [16] may arise from lack of endoscopic evaluation of the neoterminal ileum during pouchoscopy for presumed pouch failure; ileoscopic evaluation for PI typically occurs much later after symptom onset than evaluation for pouchitis, and the endoscopic and histologic findings may have irreversibly changed after this long delay because of altered ileal microflora [11].

PI may result from mucosal inflammation from reflux into the afferent limb of altered pouch microflora secondary to pouch stasis [1, 3, 5, 10, 11]. Despite a frequent association with UC, Haboubi et al. [6] suggest PI is not necessarily related to UC. While ileal inflammation can occur in patients with unoperated UC from reflux (backwash ileitis), backwash ileitis generally resolves following RPC, while PI initially presents following RPC [11, 17].

Despite its relative rarity, PI should be considered in any patient status-post IPAA and RPC who presents with IPAA complications consistent with pouchitis. Such patients should undergo pouchoscopy with afferent limb intubation. Treatment should be initiated promptly after diagnosis of PI, with addition of immunomodulator or biologic therapy as necessary. This well-documented case report illustrates the clinical, radiologic, endoscopic, and histologic findings of PI, a relatively rare and inadequately understood disease. Further investigations are needed to better understand the pathophysiology of PI.
